# Colorimetric Optode Sensor with Tripodal Ionophore for Rapid Urinary Ammonium Determination

**DOI:** 10.1021/acssensors.5c00756

**Published:** 2025-05-14

**Authors:** Fangmei Fu, XinYu Zhang, Wei Wang, Xiaojiang Xie

**Affiliations:** † Department of Chemistry, 58207 The Hong Kong University of Science and Technology, Clear Water Bay, Kowloon, Hong Kong, China; ‡ Department of Chemistry, 255310 Southern University of Science and Technology, Shenzhen 518055, China

**Keywords:** ammonia sensor, ionophore, optodes, urine, colorimetry

## Abstract

The amount of ammonium excreted in urine is a crucial indicator for assessing renal metabolic acidosis. Therefore, there is a significant demand for chemical sensors capable of accurately determining urinary ammonium concentrations with high throughput. In this study, we developed an ammonia chemical sensor based on the ion-selective optode principle. This sensor employs strategically designed ionophores and a PTFE gas-permeable film to enhance selectivity for free NH_3_. The low basicity of the chromoionophore indicator and the weak affinity of the tripodal ammonium receptor (L, log β = 4.62 ± 0.18) contributed to a markedly reduced response time, achieving detection within seconds, a significant improvement over previous sensors. A limit of detection (LOD) of approximately 15 nM was obtained in colorimetric mode. Total inorganic ammonia content in undiluted human urine was successfully determined using the sensor. Additionally, the optode was adapted into a multiwell format, offering potential for high-throughput point-of-care testing of urinary ammonium concentrations and other samples.

## Graphical abstract

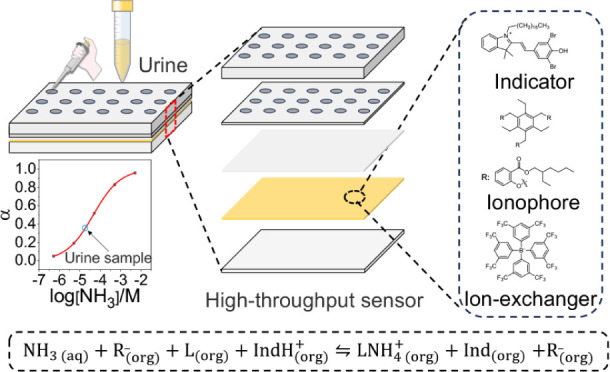

The concentration of total ammonia nitrogen (N_T_) in body fluids refers to the sum of NH_4_^+^ and free NH_3_, with the distribution determined by the pH of the environment. The pH of body fluids is typically below the p*K*_a_ of NH_4_^+^(9.3), which makes NH_4_^+^ account for ca. 99% of N_T_.^1^ Ammonium excretion plays a key role in the body’s acid–base balance, usually accounting for about two-thirds of the net renal acid excretion in the kidneys.^2^ When extrarenal metabolic acidosis is present, normal human urinary ammonium excretion can increase even by several folds.^3^ However, in patients with chronic kidney disease (CKD), declining renal function leads to decreased renal acid excretion capability.^4,5^ Low urinary ammonium excretion is one of the significant causes of CKD-related renal failure and patient mortality.^6,7^ Therefore, measuring urinary ammonium concentration allows healthcare providers to assess the condition of CKD patients in a timely manner and provide alkali therapy before the development and worsening of metabolic acidosis.^8^

However, the direct determination of urinary ammonium is not widely practiced in clinical settings due to a lack of robust sensors. The urinary anion gap (UAG) and urinary osmotic gap (UOG) are often used to indirectly estimate urinary ammonium concentration.^9,10^ Unfortunately, many reports have indicated that these methods exhibit poor correlation with urinary ammonium concentration and low consistency across multiple measurements, in addition to being time-consuming.^11–13^ Researchers have proposed using the glutamate dehydrogenase rate method, which is employed for blood ammonium, to directly determine urinary ammonium.^14,15^ However, enzyme assay methods commonly suffer from changes in experimental conditions (e.g., temperature, pH, etc.) and the presence of enzyme inhibitors. Additionally, kinetic measurements require strict control over the timing of sample addition, typically operating on a per-sample basis, which is undesirable for high-throughput analysis.

Since ammonia is a basic chemical, chemical ammonia sensors indirectly measuring pH have been proposed in analogy to carbon dioxide sensors. For example, Borisov and Klimant developed an optode based on BODIPY fluorescent dye for trace-level ammonia detection.^16^ However, hydrogels and thin-layer aqueous solutions may be prone to dehydration and are affected by osmotic pressure. These types of sensors also require improvements in response time and selectivity.

Ionophore-based sensors, including ion-selective electrodes (ISEs) and ion-selective optodes (ISOs), have shown great potential due to improved selectivity. However, the ionic radii of NH_4_^+^ and K^+^ are close (1.38 and 1.43 Å, respectively), causing the two ions to compete for binding to ionophores,^17,18^ making it challenging to enhance the selectivity of ion carriers. For instance, nonactin has a logarithmic selectivity coefficient for NH_4_^+^ over K^+^ ranging from −0.6 to −1.7,^19,20^ indicating that such sensors are not suitable for direct use in urine samples containing high levels of K^+^. Gas-permeable membranes, such as hydrophobic porous polytetrafluoroethylene (PTFE), can allow the transportation of free NH_3_ and effectively reduce the interference of hydrophilic ions. ISEs based on nonactin and gas-permeable membranes have been successfully applied in the detection of blood ammonia and environmental samples,^21,22^ although calibration of such electrodes is nontrivial.

Ion-selective optodes (ISOs), as the optical counterparts of ISEs, incorporate chromoionophores (lipophilic pH indicators) to transduce chemical recognition into optical signals.^23^ ISOs can be operated in various modes, including fluorescence, light absorption, and colorimetry.^24,25^ ISOs in the form of a hydrophobic polymeric thin film can mitigate the influence of osmotic pressure on sensor performance. However, it is critical to properly choose the combination of chromoionophore (for H^+^) and ionophore (for the target ion).

Earlier, Simon’s group proposed ISOs with nonactin and different chromoionophores to detect ammonia concentration in gas or liquid phases.^26,27^ Strangely, these sensors exhibited long response times. Upon revisiting this issue, we noticed the rather high basicity of the chromoionophores and the strong binding between nonactin and ammonium, which could kinetically limit the sensor response. In this study, we adopted carrier molecules with lower binding constants (both H^+^ chromoionophore and ammonium ionophore) to prepare a rapid-response ammonia sensor in colorimetric mode. The optode sensor was successfully used for the determination of ammonium content in undiluted urine samples. The optode sensor was further adapted to require a sample volume of just 20 μL, making it promising for POCT and high-throughput analysis.

## Experimental Section

### Reagents and Materials

Polyvinyl chloride (PVC, high molecular weight), 2-nitrophenyl octyl ether (NPOE), bis­(2-ethylhexyl) sebacate (DOS), sodium tetrakis-[3,5-bis­(trifluoromethyl)-phenyl]­borate (NaTFPB), nonactin, chromoionophores (ChI, ChIII, and ChVII), urea, uric acid, creatinine, and tri­(hydroxymethyl)­aminomethane (Tris) were purchased from Sigma-Aldrich. 3,5-Dibromo-4-hydroxystyryl-3,3-dimethyl-1-octadecyl-3H-indol-1-ium, a weakly basic chromoionophore and a tripodal ionophore (L), was custom-synthesized in the laboratory (structural characterization is provided in the Supporting Information). Uric acid, aniline, creatinine, and ammonium chloride were purchased from Bide Pharmatech in China. Polytetrafluoroethylene (PTFE) gas-permeable membrane (50 μm thick) and the enzyme-based blood ammonia kit were purchased from local providers in China. Artificial urine was prepared according to the literature.^28^ All salt solutions and buffers were prepared with deionized water with a resistivity of 18.2 MΩ·cm (Milli-Q Integral 5).

### Preparation of NH_3_ Optode Sensor

Typically, 2.0 mg of the tripodal ionophore (L), 1.0 mg of NaTFPB, and 0.4 mg of the chromoionophore were dissolved in 400 μL of THF. The cocktail was carefully pipetted onto a glass substrate and immediately cast with a knife coater, forming a 20 μm thick layer. After THF evaporation, an optode film with a thickness of about 10 μm was obtained. The optode film was washed with deionized water to release NaI salts and fully deprotonate the indicator dyes. Afterward, the film was covered with a PTFE gas-permeable membrane, and the periphery was sealed with adhesive tape. To make the high-throughput assay, additional layers made of silicone rubber (0.43 mm thick) and poly­(methyl methacrylate) (PMMA, 3 mm thick) were laser cut to form evenly distributed wells and placed on top of the gas-permeable membrane.

### Instrumentation and Measurements

UV–vis absorption spectra were measured using a UV–vis spectrometer (SPECORD 250 Plus, Analytik Jena). The multiwell silicon/PMMA layer (4.4 cm × 2.5 cm) was fabricated on the xTool P2 55W desktop CO_2_ laser cutter. Pictures of the optode sensors were taken with a digital camera (Canon EOS 5D Mark IV) using the following parameters: ISO 1000, f/2.5, 1/250 s exposure, auto white balance, and a frame rate of 1 Hz for video mode. The absorbance of the blood ammonium kit was recorded using a microplate reader (Cytation 5, BioTek). The synthesized ionophore and indicator were characterized using a 400 MHz Bruker AVANCE NEO nuclear magnetic resonance (NMR) spectrometer and a Q-Exactive electrospray ionization mass spectrometer (ESI-MS) in positive-ion mode (Thermo Fisher Scientific, USA). The electrode potential was recorded using a 16-channel precision electrochemistry interface (EMF 16, Lawson Laboratories Inc., USA). Scanning electron microscope (SEM) images were obtained using scanning electron microscopy (Regulus 8100, Hitachi).

The pictures of the optodes were analyzed using ImageJ software to retrieve the values in the red (R) and blue (B) channels. The degree of deprotonation (α) was calculated according to eq. S2, where R_P_/G_P_ and R_D_/G_D_ represent the values for the fully protonated and fully deprotonated states, respectively, measured in Tris-HCl buffer solutions containing 0 and 1 M ammonium chloride, respectively. Alternatively, for optode films measured using UV–vis spectroscopy, α was calculated based on absorbance, where A_P_ and A_D_ correspond to the absorbance of the fully protonated and fully deprotonated states determined in 0.1 M HCl and 0.1 M NaOH, respectively.

The commercial blood ammonia assay based on glutamate dehydrogenase was used according to its manual. Briefly, a calibration curve was established in 96 wells on a plate reader in pH 7.8 Tris-HCl buffer with different NH_4_Cl concentrations. The temperature was controlled at 37 °C, and absorbance at 340 nm was monitored over time.

## Results and Discussion

Figure 1 illustrates the basic structure of the ammonia optode sensor. A PVC-NPOE film, containing the tripodal ionophore for NH^4+^ ions (L) and the weakly basic chromoionophore (WB, existing between protonated HInd^+^ and deprotonated Ind^+^ forms), is knife coated onto a glass substrate. A PTFE gas-permeable film is placed above the PVC-NPOE layer. In the presence of NH_4_^+^, free NH_3_ can permeate through the PTFE into the PVC-NPOE layer due to the porous structure of the PTFE film (Figure S4), deprotonate the HInd^+^, and form a complex with L. The optimal structure of L and NH_4_^+^ complexation was calculated based on density functional theory (DFT) using the software Gaussian16. We performed a geometric optimization on the L-NH_4_^+^ complex system at the M06–2X/def2-SVP theoretical level, followed by a frequency analysis at the same theoretical level on the optimized structure, confirming its stability, as no imaginary frequencies were detected. The geometric optimization of the L-NH_4_^+^ complex reveals a precise alignment, with the three ethyl groups collectively residing on one side of the benzene ring and the trio of ester groups occupying the side opposite to the ethyl groups. The distance between each hydrogen atom of the ammonium ion and the nearest oxygen atom is between the sum of the van der Waals radii of O and H (272 pm) and the covalent bond length of O–H (96 pm). This arrangement enables selective recognition of NH_4_^+^ through the formation of hydrogen bonds between the carbonyl oxygen atoms of the ester groups and the hydrogen atoms of NH_4_^+^.

**Figure 1:**
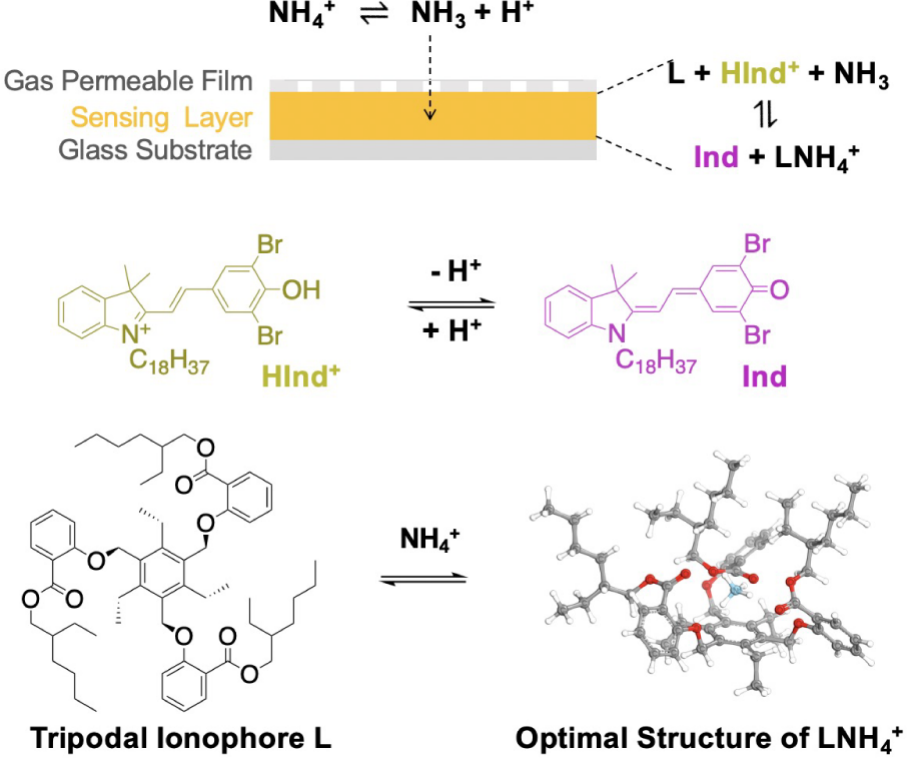
Schematic illustration of the structure, composition, and mechanism of the NH_3_ optode sensor composed of a glass substrate, a PVC-NPOE sensing layer, and a PTFE gas-permeable film. The sensing components include the chromoionophore (WB) and the tripodal ionophore for NH_4_^+^ ions (L). Computationally optimal complex (LNH_4_^+^) was calculated with DFT at the M06–2X/def2-SVP level.

Additionally, we determined the stability constant (log β) of the complex LNH_4_^+^ in a PVC-NPOE membrane using the sandwich membrane method,^29^ obtaining a value of 4.62 ± 0.18. Compared to the widely recognized ammonium ion carrier nonactin, L has a much weaker affinity for ammonium ions.^30^ This is crucial for achieving a fast-responding sensor, a similar observation noted by Simon et al. in previous studies.^27^

The sensing process can be represented by eq 1, where L denotes the ionophore, R^–^ denotes the TFPB anion, Ind represents the deprotonated chromoionophore, LNH_4_^+^ represents the complex formed by the ammonium ion and the carrier L, and aq and org represent the aqueous and organic phases, respectively.

1 $${\mathrm{NH}}_{3\left(\mathrm{aq}\right)}+R_{\left(\mathrm{org}\right)}^{\text{\_}}+L_{\left(\mathrm{org}\right)}+{\mathrm{HInd}}_{\left(\mathrm{org}\right)}^{+}⇋{\mathrm{LNH}}_{4\left(\mathrm{org}\right)}^{+}+{\mathrm{Ind}}_{\left(\mathrm{org}\right)}+R_{\left(\mathrm{org}\right)}^{\text{\_}}$$

The concentration of free NH_3_ in the aqueous phase was solved and expressed in eq 2, where square brackets denote concentrations, K represents the overall equilibrium constant, R_T_, L_T_, and Ind_T_ are the total concentrations of TFPB, L, and WB, respectively, and α represents the degree of deprotonation of the chromoionophore. It should be noted that the free NH_3_ concentration in the aqueous phase depends on the sample pH and the total concentration of the ammonium species N_T_, which is expressed in eq 3 derived from the Henderson–Hasselbach equation.

2 $$\left[{\mathrm{NH}}_{3}\right]=\frac{\alpha \left[R_{T}^{-}-\left(1-\alpha \right){\mathrm{Ind}}_{T}^{+}\right]}{K\left(1-\alpha \right)\left[\left(1-\alpha \right){\mathrm{Ind}}_{T}^{+}-R_{T}^{-}+L_{T}\right]}$$

3 $$\mathrm{pH}=\mathrm{pKa}+\log\frac{\left[{\mathrm{NH}}_{3}\right]}{[N_{T}]-[{\mathrm{NH}}_{3}]}$$

Under the same N_T_ value, increasing the solution pH will generate more NH_3_, making it easier to detect by the sensor. Figure 2a shows the color of the sensor response at three different pH conditions (7.0, 8.0, and 9.0 Tris-HCl buffer). The optode appears light yellow-green in the initial protonation state. As N_T_ increases, the chromoionophore gradually deprotonates (α increases) and turns reddish-purple. Figure 2b shows the relationship between α and the concentration of free NH_3_ in the solution. The data under different pH values were fitted by the same theoretical curve, proving that the optode sensor indeed responds to free NH_3_. The lower detection limit (LOD) of the sensor composed of L and our chromoionophore was determined to be 15.5 nM (based on 3 times the standard deviation above background intensity). The sensor showed a wide dynamic response range, covering the common range of free NH_3_ in urine (assuming a typical urine pH of 4.5–8 with N_T_ from 10 to 70 mmol/L for healthy adults). However, a potential limitation is that a lower sample pH reduces the free NH_3_ concentration in solution, limiting the amount available for detection by the sensor.

**Figure 2:**
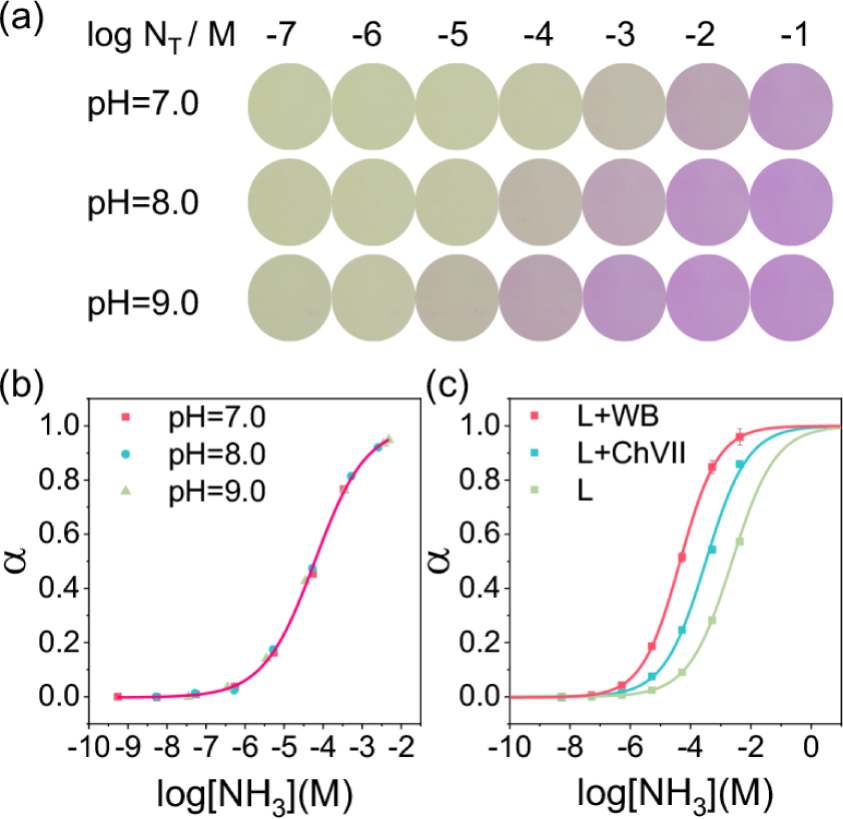
(a) The color of the sensor equilibrated with different N_T_ at three different pH conditions (7.0, 8.0, and 9.0); (b) calibration curve of the ammonia sensor in different pH buffers; (c) response curves of 3 different ammonia sensors: L + WB, L + ChVII, and L (pH = 8.0). All solutions were prepared with a 50 mM Tris-HCl buffer.

Table 1 summarizes the performance of the sensor without or with different chromoionophores. The ultrahigh sensitivity of our sensor for low-concentration ammonia is ascribed to two aspects: (1) L can effectively bind ammonium ions, and (2) the indicator has a weak affinity for protons (p*K*_a_ = 10.8). When L is removed from the PVC-NPOE membrane, the sensor’s response range shifts toward the high concentration range by about 1.8 units, becoming less sensitive, and the detection limit increases to about 0.33 μM. Second, if the synthesized chromoionophore is replaced with the Nile Blue series commonly used in ISOs (such as ChVII), the sensor’s response range will still shift to the right by about 1 unit, and the detection limit will become approximately 61.1 nM. The p*K*_a_ of our chromoionophore in PVC-NPOE is indeed lower than that of ChVII (p*K*_a_ = 11.7 in PVC-NPOE, the least basic chromoionophore derived from Nile Blue).^31^

**Table 1:** Comparison of LOD and the Free NH_3_ Concentration Corresponding to α = 0.5 of the Three Sensors

Sensor	LOD (M)	log[NH_3_ (M)] α = 0.5
L + WB	1.55 × 10^–8^	–4.40
L + ChVII	6.11 × 10^–8^	–3.49
L	3.34 × 10^–7^	–2.62

The ammonia optode sensor exhibited a very fast response. As shown in Figure 3a, a response time (t_95_) of 2.8 min at low N_T_ concentrations of 10^–6^–10^–5^ M was observed. As the concentration increased, the response time further decreased, with a t_95_ of only 33 s for concentrations from 10^–3^ to 10^–2^ M. When the solution was changed from 0.1 M NH_4_Cl to a blank solution of 50 mM Tris-HCl, the sensor fully recovered to its initial state within 4.5 min, also proving that its response to NH_3_ is reversible. It is worth noting that due to the fast recovery, the solution was replaced twice with a blank for α return to the initial state in Figure 3a.

**Figure 3:**
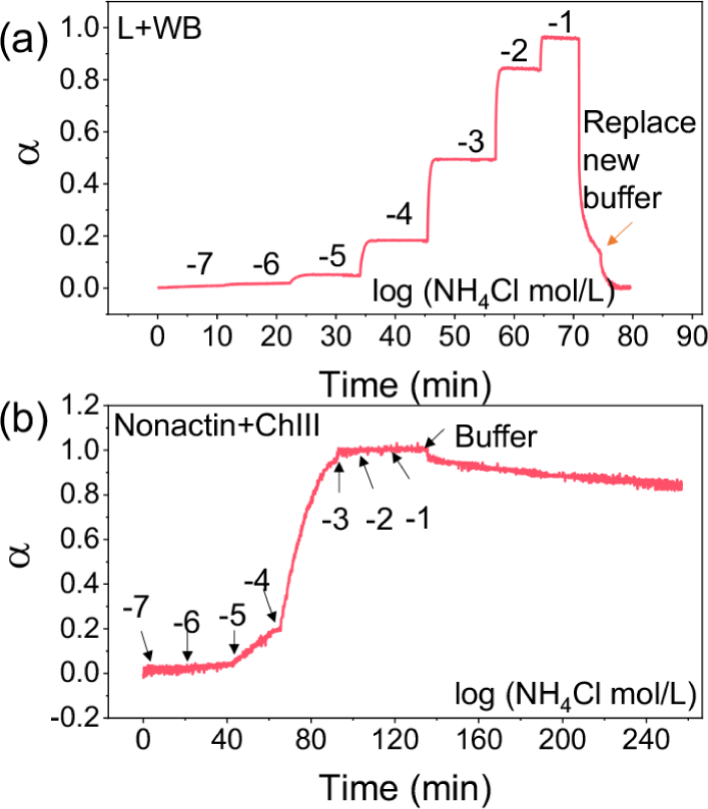
Dynamic response of ammonia optode sensor containing L + WB (a) and nonactin + ChIII sensor (b) in pH = 8.0 50 mM Tris-HCl buffer with different values of logN_T_, as indicated. The black arrows indicate the time to change the sample concentration. The orange arrow indicates that the sample was replaced with blank buffer one more time.

In stark contrast, when L was replaced with nonactin, the response time significantly increased, and the reversibility became poor. Figure 3b shows the real-time response of a sensor containing nonactin and another commercial chromoionophore (ChIII) to different concentrations of N_T_.

First, it was difficult to reach equilibrium at low concentration ranges, even from 10^–4^ to 10^–3^ M, and stability was not achieved even after 20 min. Second, when the solution changes from 0.1 M NH_4_Cl to a blank solution of 50 mM Tris-HCl, the signal only recovers to about 16% within 2 h. Similar phenomena persist when ChIII is replaced with ChVII and ChI (Figure S5).

We speculate that the prolonged response time here is mainly related to the high basicity of the chromoionophore and the strong binding affinity of nonactin for ammonium ions. On one hand, the binding constant (logβ) of nonactin with ammonium is about 14.1^30^, which is several orders of magnitude higher than that of our L. This will lead to smaller ammonium ion concentration gradients in the film and make mass transport more reliant on ionophores (diffusing slower). On the other hand, the chromoionophore (such as ETH 5294) is more basic than NH_3_, making it difficult to form NH_4_^+^ (making NH_4_^+^ concentration very low in the film) and taking longer to get NH_4_^+^ complexed by ionophore.

Urine samples contain a complex mixture of components, primarily inorganic salts and metabolic waste products, such as urea, uric acid, and creatinine. These waste products contribute roughly 42% and 58% of urine’s solid matter, respectively, with urea alone accounting for around half.^28^ Consequently, a sensor for free NH_3_ in urine needs exceptional selectivity to avoid interference from high salt ion and organic amine concentrations. Figure 4a shows the remarkable selectivity of our optode sensor containing L and the chromoionophore. Even when concentrations of Na^+^, K^+^, Mg^2+^, and Ca^2+^ (0.1 M) are 100 times higher than the total ammonium concentration (N_T_ = 1 mM), the sensor’s response signal (α) to free NH_3_ (generated from ammonium hydrolysis) is still 20 times higher. This indicates the crucial role of the PTFE ion barrier in mitigating interference. While uric acid and urea caused negligible interference, creatinine and aniline, with 1 mM concentration, resulted in a relatively higher interference. The results indicate that such neutral biomolecules could potentially pass through the PTFE membrane. Fortunately, urine is typically weakly acidic, creatinine exists as cations under such conditions, and aniline is not present in urine at high concentrations. As shown in Figure 4b, ions and organic amines in artificial urine (AU) with pH = 4.0–7.4 did not interfere with the response of sensor. However, urine’s broad pH distribution may bring additional issues to the sensor, especially when the pH is very low (e.g., pH 4.5, Figure 4b). In the case of urine samples from CKD patients, where the N_T_ is typically even lower, selectivity issues should be separately assessed.

**Figure 4:**
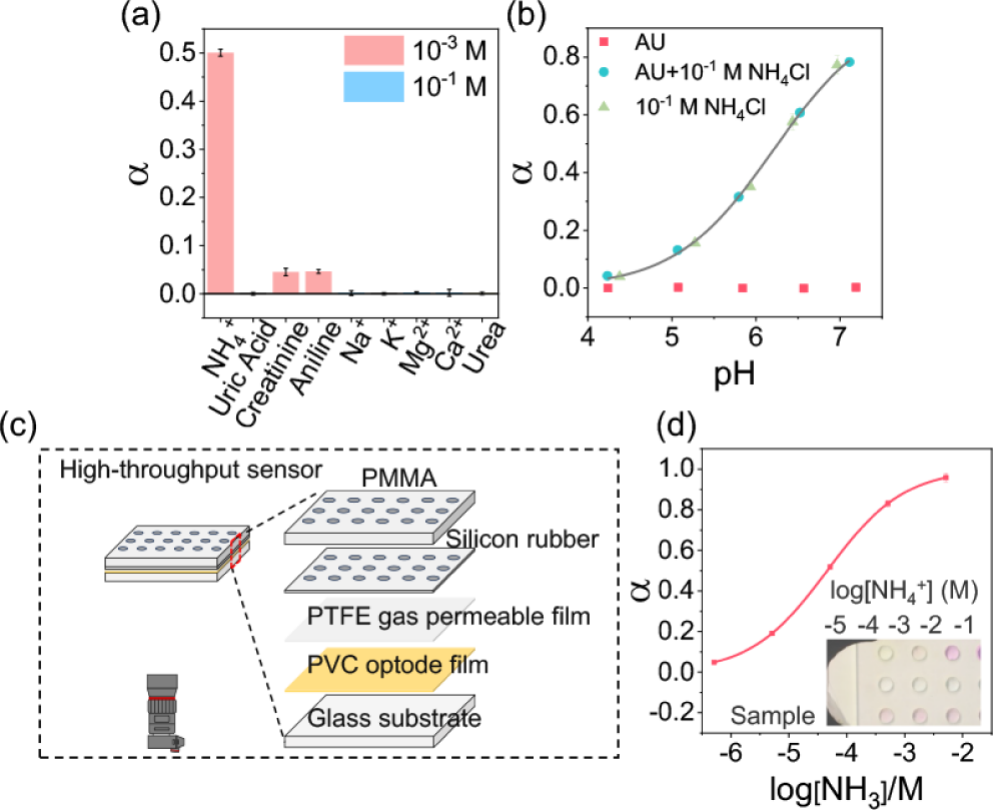
(a) Response of the ammonia sensor containing L and WB to different analytes with indicated concentrations; (b) influence of sample pH artificial urine (AU) background on the response of the ammonia sensor; (c) design of high-throughput sensing mode; (d) the response of sensor (α) in high-throughput mode as a function of logarithmic free NH_3_ concentration (pH = 8.0, 50 mM Tris-HCl buffer).

As an application, the optode sensor was used to determine the total ammonium concentration in undiluted human urine. The N_T_ value was determined to be 29.6 ± 0.1 mM, which is within the range of healthy adult samples. As a control method, we also evaluated the same sample with the glutamate dehydrogenase assay, which is a method previously used for blood ammonium determination (see Experimental Section and Figure S3 for more details). The control method reported a value of 29.4 ± 0.1 mM, which is close to the results from the optode sensor.

In addition, we attempted to develop a multiwell sensor for high-throughput analysis of real samples. As shown in Figure 4c, the PVC-NPOE optode is 4.4 × 2.5 cm in size. On top of the gas permeable membrane, a silicone pad and a PMMA cover containing the wells were placed. Each sample well had a volume capacity of 20 μL. Figure 4d shows the response curve determined in the high-throughput sensor. It is worthwhile to mention that with the small urine sample volume, pH change may become an issue during the operations. Also, considering the low free ammonia concentration at acidic pH, it is worth adjusting the pH. Here, we mixed the samples with a pH buffer (pH 7.0 Tris-HCl 50 mM) in a 1:1 ratio. On the multiwell plate, a urinary ammonium concentration of 3.2 ± 0.2 mM was determined in a volunteer urine sample, which is close to a value of 2.9 ± 0.1 mM obtained from the glutamate dehydrogenase assay.

## Conclusion

We presented a novel NH_3_ sensor based on ion-selective optode principle for use in the aqueous phase. The optode sensing layer contained a chromoionophore with a strategically low p*K*_a_ and a tripodal ammonium ion carrier, enabling the rapid detection of free NH_3_ in urine samples. This design also improved the response speed by lowering both the chromoionophore’s p*K*_a_ and the ammonium affinity of the ion carrier, surpassing the limitations of previous sensors reliant on nonactin and Nile Blue chromoionophores. Furthermore, a gas-permeable PTFE membrane acted as an ion barrier, significantly enhancing the sensor’s selectivity for free NH_3_. The sensor design holds promise for future development into a multiwell plate format. Such a format would facilitate the establishment of a calibration curve and the simultaneous measurement of multiple samples on a single plate, minimizing sample volume and enabling high-throughput analysis.

## Supplementary Material



## Notes

The synthetic routes, ^1^H NMR spectra, ^13^C NMR spectra, and mass spectra of WB and L; determination of urinary ammonium by glutamate dehydrogenase rate method; SEM images of PTFE gas-permeable film; the response time of sensors nonactin + ChVII and nonactin + ChI; detailed computational information on the optimal structure of L and NH_4_^+^ complexation (PDF)
