# Correction to “Colorimetric Optode Sensor with Tripodal Ionophore for Rapid Urinary Ammonium Determination”

**DOI:** 10.1021/acssensors.5c02449

**Published:** 2025-07-22

**Authors:** Fangmei Fu, XinYu Zhang, Wei Wang, Xiaojiang Xie

The correct Figure 2c is shown below, where the green trace should be labeled WB, indicating an optode sensor containing only our synthesized chromoionophore WB.

**Figure 2:**
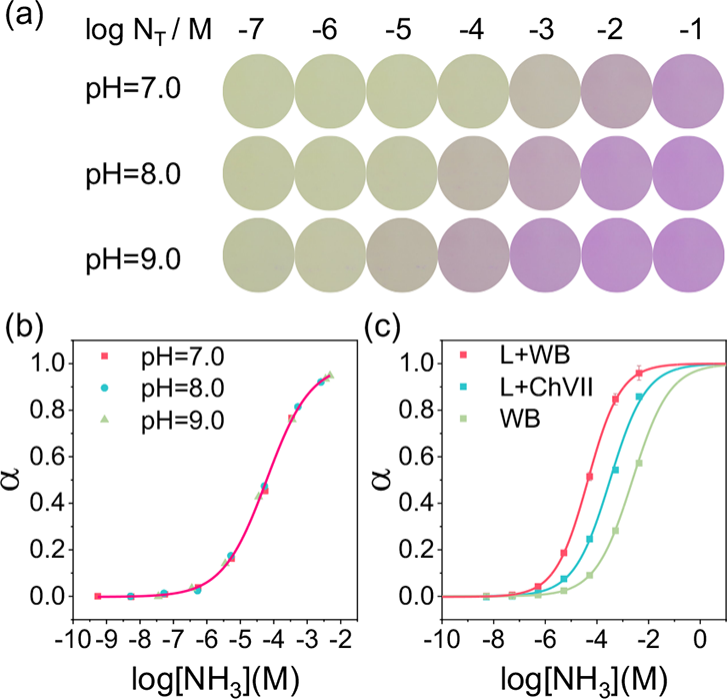
(a) The color of the sensor equilibrated with different N_T_ at three different pH conditions (7.0, 8.0, and 9.0); (b) calibration curve of the ammonia sensor in different pH buffers; (c) response curves of 3 different ammonia sensors: L + WB, L + ChVII, and WB (pH = 8.0). All solutions were prepared with a 50 mM Tris-HCl buffer.

Corresponding to Figure 2c, the label for the same optode in Table 1 also should be WB.

**Table 1:** Comparison of LOD and the Free NH_3_ Concentration Corresponding to α = 0.5 of the Three Sensors in Figure 2c

Sensor	LOD (M)	log[NH_3_ (M)] α = 0.5
L + WB	1.55 × 10^–8^	–4.40
L + ChVII	6.11 × 10^–8^	–3.49
WB	3.34 × 10^–7^	–2.62

The inset picture of Figure 4d was truncated during formatting. The correct Figure 4 is shown below.

**Figure 4:**
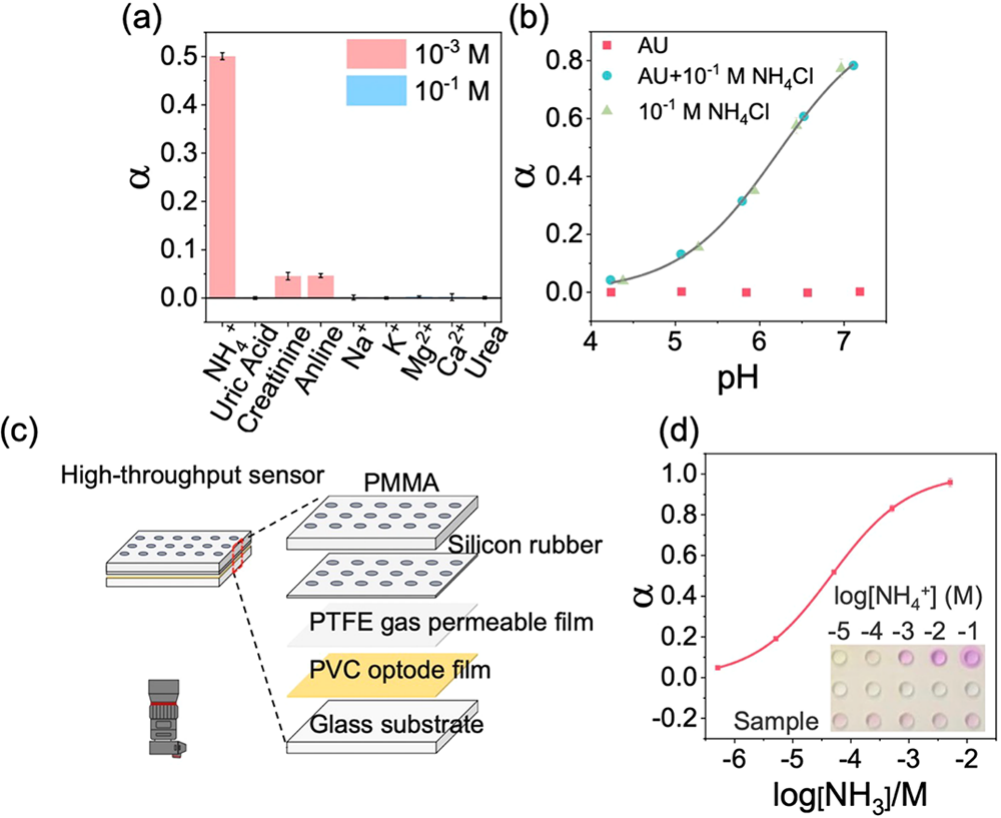
(a) Response of the ammonia sensor containing L and WB to different analytes with indicated concentrations; (b) influence of sample pH artificial urine (AU) background on the response of the ammonia sensor; (c) design of high-throughput sensing mode; (d) the response of sensor (α) in high-throughput mode as a function of logarithmic free NH_3_ concentration (pH = 8.0, 50 mM Tris-HCl buffer).

These changes do not affect the scientific conclusions of the study but require clarification for accuracy. We sincerely apologize for these oversights and any confusion to readers.

