# Supplementary material for: Colorimetric Optode Sensor with Tripodal Ionophore for Rapid Urinary Ammonium Determination
Source: ACS Sens. 2025 May 14;10(5):3757–62. doi: 10.1021/acssensors.5c00756 (PMC12105079; doi:10.1021/acssensors.5c00756)
Supplement: Supplementary file 1 [file se5c00756_si_001.pdf]

Supporting Information

Colorimetric Optode Sensor with Tripodal Ionophore for Rapid Urinary Ammonium Determination

Fangmei Fu[#], XinYu Zhang[#], Wei Wang[§], and Xiaojiang Xie[§]*

[§]Department of Chemistry, The Hong Kong University of Science and Technology, Clear Water Bay, Kowloon, Hong Kong, China.

[#] Department of Chemistry, Southern University of Science and Technology, Shenzhen, 518055, China

*E-mail: xiexj@ust.hk

Contents

Synthesis of chromoionophore (WB) and tripodal ionophore (L) Page S2

Figure S1

Figure S2

Determination of urinary ammonium by glutamate dehydrogenase rate method Page S4

Figure S3

Figure S4

Figure S5

Eqn. S1

Eqn. S2

Computational details Page S7

1. Synthesis of chromoionophores and tripodal ionophore (L)

1.1 Synthesis of the chromoionophore WB used in this work

The intermediate 2,3,3-trimethyl-1-octadecyl-3H-indole-1-iodine ($C_{29}H_{50}IN$) was synthesized. The 2,3,3-trimethylindole ($C_{11}H_{13}N$) (1.0 g, 6.28 mmol) and iodooctadecane ($C_{18}H_{37}I$) (3.58 g, 9.42 mmol) were refluxed in 4 mL acetonitrile at 90℃ for 14 h, cooled to room temperature, added cold ether to improve the precipitation, filtered, and washed several times with cold ether. The yield was 71%. ^{1}H NMR (600 MHz, Chloroform-*d*) δ 7.64 (dd, *J* = 6.0, 2.5 Hz, 1H, Ar-H), 7.59 (d, *J* = 3.7 Hz, 3H, Ar-H), 4.69 (t, *J* = 7.8 Hz, 2H, CH_2-H), 3.13 (s, 3H, CH_3), 1.94 (p, *J* = 7.8 Hz, 2H, CH_2-H), 1.67 (s, 6H, $CH_3 \times 2$), 1.46 (p, *J* = 7.5 Hz, 2H, CH_2-H), 1.37 (p, *J* = 6.9 Hz, 2H, CH_2-H), 1.25 (d, *J* = 6.3 Hz, 26H, CH_2-H × 13), 0.88 (t, *J* = 6.9 Hz, 3H, CH_3).^{13}C NMR (151 MHz, $CDCl_3$) δ 195.68, 141.82, 141.15, 130.26, 129.64, 123.47, 115.44, 54.79, 50.42, 32.03 (Two), 29.81, 29.76, 29.73 (Three), 29.68 (Two), 29.57, 29.46, 29.44, 29.25, 28.07, 26.96, 23.34 (Two), 22.82, 22.79, 17.26, 14.22, 0.10. HR-ESI-MS: m/z calcd for $C_{29}H_{50}N^+$, 412.7225; found, 412.3940.

The final product (E) -2- (3,5-dibromo-4-hydroxystyryl) -3,3-dimethyl-1-octadecyl-3H-indole-1-iodine ($C_{36}H_{52}Br_2INO$) was synthesized. The 2,3,3-trimethyloctadecylindole iodide ($C_{29}H_{50}IN$) (0.5393 g, 1 mmol) and 3,5-dibromo-4-hydroxybenzaldehyde ($C_7H_4Br_2O_2$) (0.2779 g, 1 mmol) were refluxed in 15 mL ethanol with 1 drop of piperidine as catalyst under nitrogen atmosphere at 85℃ for 18 h. The final product was purified by column chromatography purification, and the yield was 67 %. ^{1}H NMR (600 MHz, DMSO-*d*$_6$) δ 8.52 (s, 1H, Ar-H), 8.11 (s, 1H, Ar-H), 8.03 (d, *J* = 14.7 Hz, 1H, CH=CH-H_1), 7.63 (d, *J* = 7.0 Hz, 1H, CH=CH-H_2), 7.47 – 7.39 (m, 2H, Ar-H), 7.29 (td, *J* = 7.1, 1.5 Hz, 1H, Ar-H), 6.78 (d, *J* = 14.8 Hz, 1H, Ar-H), 4.31 (t, *J* = 7.2 Hz, 2H, CH_2), 1.72 (q, *J* = 7.2 Hz, 2H, CH_2), 1.67 (s, 6H, $CH_3 \times 2$), 1.39 – 1.28 (m, 4H, CH_2), 1.26 – 1.16 (m, 26H, $CH_2 \times 13$), 0.85 (t, *J* = 7.0 Hz, 3H, CH_3). ^{13}C NMR (151 MHz, DMSO) δ 196.38, 141.86, 141.03, 129.41, 128.93, 123.50, 115.43, 54.13, 47.55, 31.25 (Three), 28.99 (Five), 28.96 (Three), 28.87, 28.76, 28.66, 28.56, 27.21, 25.86, 22.05 (Three), 22.00 (Three), 13.92 (Three). HR-ESI-MS: m/z calcd for $C_{36}H_{52}Br_2NO^+$, 674.3290; found,674.2400.

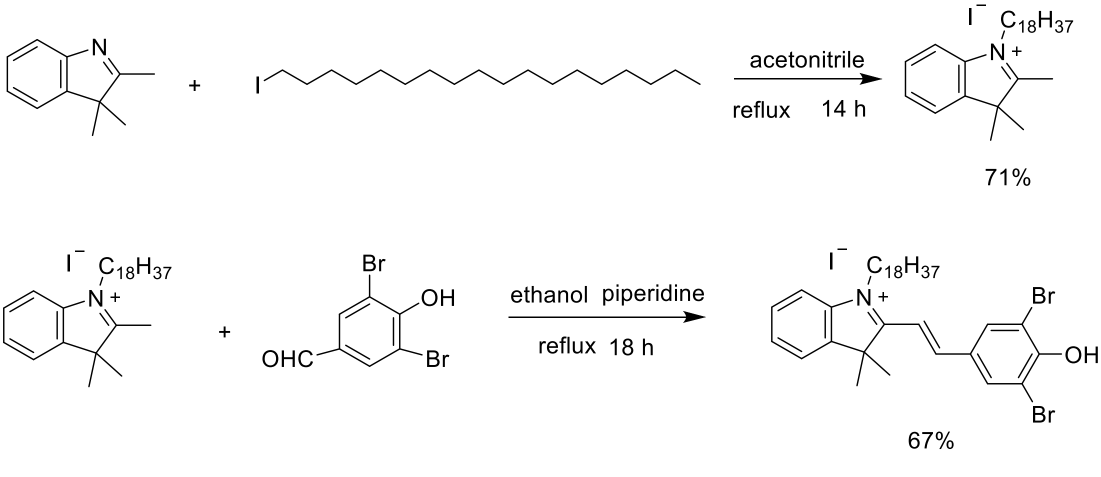

Figure S1. The synthetic route of the chromoionophore used in this work.

1.2 Synthesis of the ammonia ionophore L

Synthesis of tris (2-ethylhexyl) -2,2′2 ″- (((2,4,6-triethylbenzene-1,3,5-triyl) tris (methylene)) tris (oxy)) tribenzoate ($C_{60}H_{84}O_9$), 2-ethylhexyl 2-hydroxybenzoate ($C_{15}H_{22}O_3$) (2.24 g, 8.93 mmol) and anhydrous K_2CO_3 refluxed in 10 ml acetonitrile at 90 °C for 6 min, 1,3,5-tris (bromomethyl) -2,4. 6-Triethylbenzene ($C_{15}H_{21}Br_3$) (661.58 mg, 1.50 mmol) was dissolved in 35 ml acetonitrile (poor solubility, light yellow solution) and added to the above reflux solution at 100 °C for 29 h. The reaction mixture was extracted with water and dichloromethane, and purified by silica gel column chromatography (ethyl acetate:petroleum ether=1:70). After evaporation of the solvent, a colorless viscous liquid was obtained with a yield of 51 %.^{1}H NMR (400 MHz, $CDCl_3$， 298 K) δ7.78 (dd, J = 7.7, 1.8 Hz, 3H, Ar-H), 7.56–7.46 (m, 3H, Ar-H), 7.19 (d, J = 8.3 Hz, 3H, Ar-H), 7.03 (t, J = 7.5 Hz, 3H, Ar-H), 5.13 (s, 6H, CH_2), 4.17–4.02 (m, 6H, CH_2), 2.91–2.80 (m, 6H, CH_2), 1.55–1.45 (m, 3H, CH_3), 1.26–1.08 (m, 30H, CH_2), 0.89–0.81 (m, 9H, CH_3), 0.72 (t, J = 7.4 Hz, 9H, CH_3). ^{13}C NMR (101 MHz, $CDCl_3$) δ 166.89 (Two), 158.26 (Two), 146.72 (Two), 133.21 (Three), 131.81 (Three), 130.53 (Two), 121.81 (Two), 120.54 (Two), 113.27 (Two), 66.97 (Three), 65.33 (Two), 38.82 (Four), 30.32 (Four), 29.11 (Four), 23.59 (Four), 23.15 (Two), 23.06 (Five), 16.40 (Three), 14.23 (Five), 11.01 (Four). HR-ESI-MS: m/z calcd for $[M+Na]^+$, 971.6008; found, 971.6000.

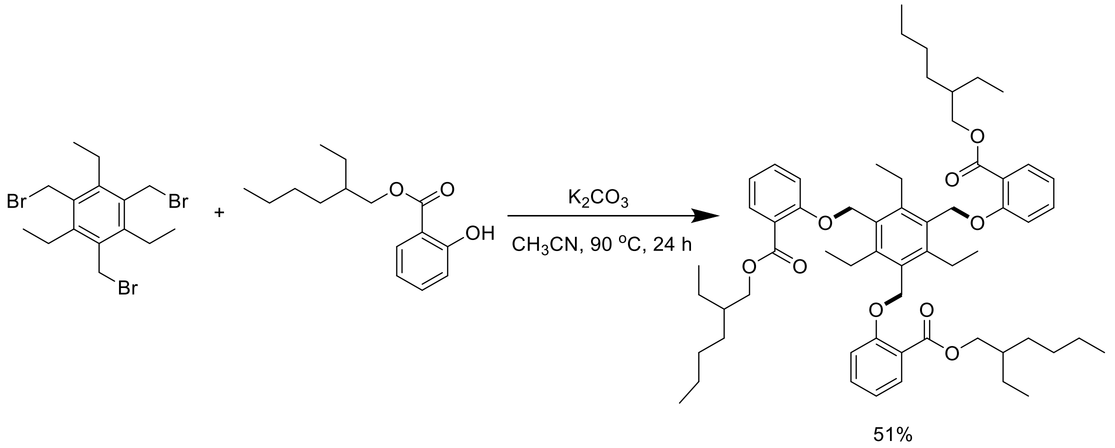

Figure S2. The synthetic route of the ammonia ionophore used in this work.

2. Determination of urinary ammonium by glutamate dehydrogenase rate method

Glutamate dehydrogenase rate method is a commonly used method for the detection of ammonium in body fluids. The principle of this method is shown in the reaction Eqn. S1. NH_4^+ reacts with α-ketoglutaric acid (KGA) and reduced nicotinamide adenine dinucleotide phosphate (NADPH) under the catalysis of L-glutamate dehydrogenase (GDH) to form L-glutamic acid (L-Glu) and oxidized nicotinamide adenine dinucleotide phosphate ($NADP^+$).

$$KGA + NH_4^+ + NADPH \rightarrow L\text{-}Glu + NADP^+ + H_2O \qquad \text{(Eqn. S1)}$$

Theoretically, the decrease rate of absorbance of NADPH at 340 nm per minute is proportional to the content of urinary ammonium. The experimental results show that (Figure S3a), after adding the sample, the decrease

in absorbance of NADPH at 340 nm per minute is not perfectly linear, but the decrease in absorbance between the 2nd minute and the 5th minute can be approximately regarded as linear, and the correlation coefficient R^2=0.994. As shown in Figure S3b, the standard curve between the two-absorbance difference ΔA at 340 nm and the concentration of NH_4Cl at 2 and 5 minutes of incubation with the enzyme-containing working reagent and the standard NH_4Cl solution was established (R^2=0.999). It is worth noting that this method is more suitable for blood ammonia detection, and the urine ammonium content often exceeds the range of this method Figure S3c. After many experiments, it is recommended to dilute the urine by 20 times before detection is more accurate.

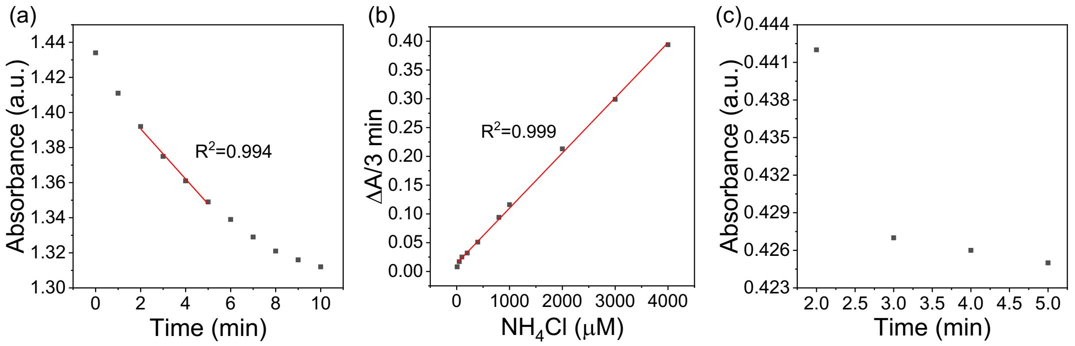

Figure S3. (a) The decreasing trend of absorbance at 340 nm per minute after the addition of the enzyme-containing working reagent to the sample; (b) The standard curve between the two absorbance difference ΔA and the concentration of NH_4Cl at 340 nm at the 2nd and 5th minutes of incubation with the enzyme-containing working reagent and the standard NH_4Cl solution; (c)The decreasing trend of absorbance at 340 nm between the 2nd minute and the 5th minute of co-incubation of undiluted urine with enzyme-containing working reagents.

The degree of deprotonation (α) was calculated according to the Eqn. S2:

$$\alpha = \frac{R/G - R_P/G_P}{R_D/G_D - R_P/G_P} \qquad \text{(Eqn. S2)}$$

where R_P/G_P 和 R_D/G_D respectively represent values for the fully protonated and fully deprotonated states, measured in pH=8.0 50 mM Tris-HCl buffer solutions containing 0 and 1 M NH_4Cl, respectively.

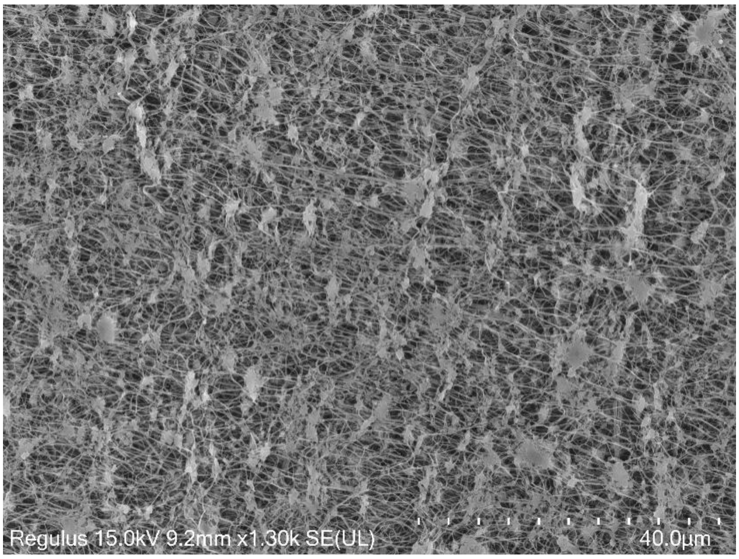

Figure S4. SEM of PTFE gas permeable film

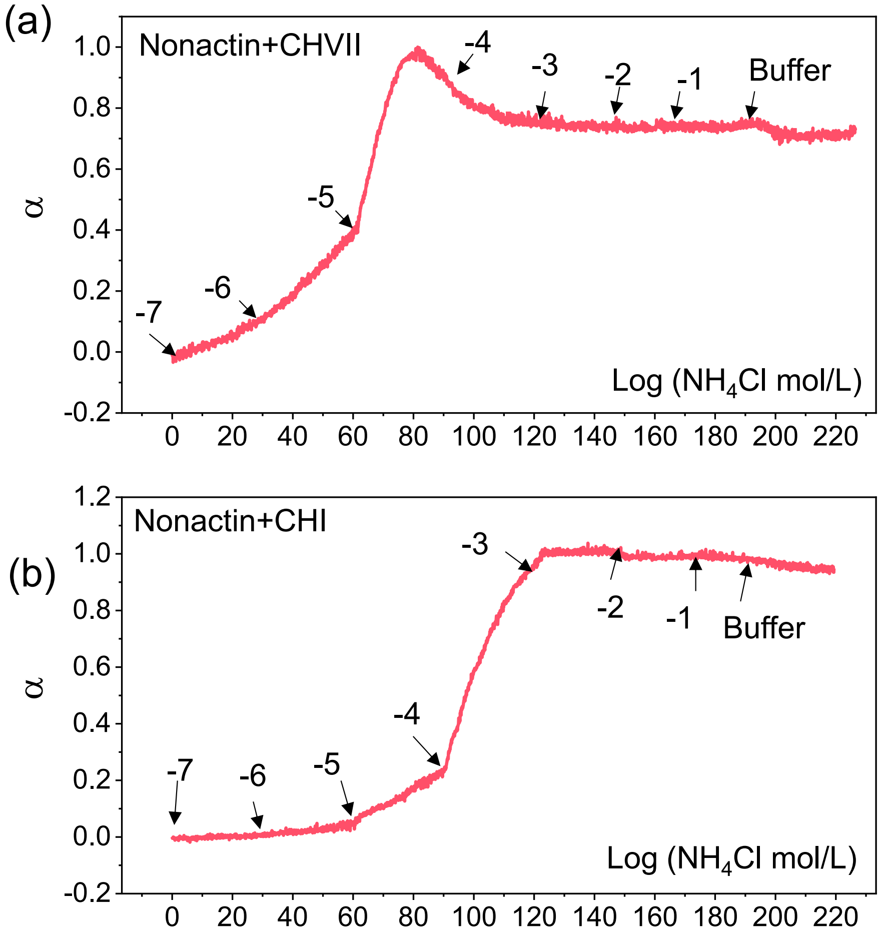

Figure S5. Dynamic response of the nonactin+ChVII sensor (a) and the nonactin+ChI sensor (b) at different concentrations of N_T. $\boldsymbol{\alpha}$ represents the deprotonated degree of the chromoionophore.

3. Computational details

All DFT calculations were carried out with the Gaussian **16** program. [1] The geometry optimizations were conducted using the M06-2X functional [2][3].Def2-SVP basis set [4] was used for all the atoms.To confirm whether each optimized stationary point is an energy minimum or a transition state, vibrational frequencies were computed at the same level of theory as for the geometry optimizations.

The 3D diagrams of computed species were generated using IBO view v20211019-RevA. [5]

Cartesian coordination of L-NH_4+

C	0.63522902	-2.76004362	1.38748043
C	0.09005050	-3.15971585	0.15617507
C	-1.30217165	-3.26940264	-0.00771591
C	-2.14371277	-3.03656067	1.09498600
C	-1.61273861	-2.64827241	2.33831257
C	-0.21524514	-2.55111528	2.48857437
C	2.13252549	-2.56249397	1.52414124
C	2.88757047	-3.83344214	1.92970439
C	-2.54526683	-2.34099968	3.49350722
C	-2.92171217	-3.58442519	4.30343930
C	-1.88281517	-3.67840775	-1.34908639
C	-1.98926228	-5.19885211	-1.50704662
C	0.41704212	-2.18404510	3.81583794
C	-3.63595022	-3.14668053	0.88347482
C	1.01561023	-3.46133693	-0.99642242
C	-5.99189606	-1.38278278	-2.83301312
C	-7.09005982	-2.22380886	-2.65383728
C	-7.17824710	-3.00851868	-1.50557225
C	-6.18392109	-2.96199334	-0.52673949
C	-5.09630455	-2.10670983	-0.70006297
C	-5.00540958	-1.31064225	-1.85241638
C	-3.75834442	-0.50500842	-2.05223144
O	-3.48776136	0.49881453	-1.23370495
O	-2.96703034	-0.76616967	-2.93275772
O	-4.06998224	-1.98711981	0.17841891
C	-4.35082043	0.94404521	-0.17477483
C	4.27911198	-1.13509160	-3.55329227
C	4.43766185	-2.18101826	-4.45982575
C	3.58234394	-3.27955185	-4.39827973

C	2.55633912	-3.33157619	-3.45553053
C	2.38575114	-2.27151457	-2.56418828
C	3.26408028	-1.18010242	-2.59593811
C	3.06682476	-0.05703093	-1.61697370
O	3.98262641	0.10877540	-0.66405804
O	2.12885487	0.69532241	-1.67238910
O	1.38611146	-2.23231184	-1.63223698
C	4.99581470	-0.85261065	-0.34284353
C	-1.80783115	1.64732987	3.43339399
C	-2.08198681	1.96283856	4.75907697
C	-1.35226049	1.35010371	5.78054362
C	-0.36176424	0.42117283	5.47505541
C	-0.07979884	0.10876681	4.14346585
C	-0.79001992	0.73978891	3.11347093
C	-0.58554174	0.42107867	1.66866718
O	0.62210850	0.42456563	1.12959172
O	-1.53923430	0.17907275	0.95368165
O	0.87311216	-0.82647890	3.86335668
C	1.72976558	1.15440385	1.69296923
H	2.52398015	-2.17983942	0.56952510
H	2.32554196	-1.76438968	2.25403101
H	3.96765226	-3.64203306	1.99915815
H	2.54963336	-4.20705915	2.90661768
H	2.73274023	-4.64825797	1.20813999
H	-2.09078526	-1.59466009	4.15774332
H	-3.44728780	-1.84911287	3.10323425
H	-3.60525192	-3.32978934	5.12468824
H	-3.40991753	-4.34153541	3.67234069
H	-2.02757987	-4.05692316	4.73614820
H	-2.86988804	-3.22347180	-1.49463036
H	-1.27326430	-3.26032569	-2.16387872
H	-2.42258099	-5.46252476	-2.48113406
H	-1.00535607	-5.68357961	-1.42430527
H	-2.62423117	-5.63435856	-0.72210322
H	-0.26427172	-2.36941617	4.65775157
H	1.31553417	-2.78734278	3.98621398
H	-3.87205719	-4.03928794	0.28400138

H	-4.18319859	-3.23585994	1.83084534
H	1.93491915	-3.95777318	-0.65280217
H	0.53221911	-4.12573974	-1.72262770
H	-5.89139244	-0.78193688	-3.73831677
H	-7.86849585	-2.27461596	-3.41476626
H	-8.03244803	-3.67221068	-1.36522941
H	-6.25855477	-3.57989173	0.36847720
H	-3.87818773	0.61827929	0.76066909
H	-5.33642007	0.46795032	-0.27215460
H	4.94260113	-0.26933003	-3.59271809
H	5.22699326	-2.13863423	-5.21011122
H	3.70553218	-4.10695694	-5.09801989
H	1.88542562	-4.18943415	-3.42669378
H	4.96220838	-0.94504342	0.75454663
H	4.74037592	-1.82971924	-0.77918196
H	-2.37128824	2.10990130	2.62042465
H	-2.86342637	2.68412763	4.99852363
H	-1.56054922	1.59414219	6.82306465
H	0.21002038	-0.08076073	6.25679261
H	1.94066061	0.77867144	2.70285297
H	2.56373710	0.88883126	1.03244955
N	-0.57484316	-0.05751676	-1.62765109
H	0.04875840	-0.88093066	-1.60832744
H	-1.02904279	0.04107530	-0.68680479
H	0.04010150	0.74531962	-1.80053646
H	-1.31901529	-0.16443518	-2.34312038
C	1.50999207	2.66520351	1.69406562
H	0.74820253	2.89819318	2.45970383
C	0.99464654	3.16973277	0.34255705
H	1.67947583	2.83988497	-0.45646640
H	0.02530186	2.68379272	0.12499992
C	0.79423163	4.68050793	0.28099518
H	1.75747686	5.19853581	0.42262792
H	0.15572297	5.00063647	1.12474046
C	0.17601014	5.14474871	-1.03490493
H	0.84274287	4.85562532	-1.86432729
H	-0.76874734	4.59929189	-1.20172217

C	-0.08277581	6.64592993	-1.07494380
H	0.84951914	7.21072223	-0.92651322
H	-0.51429271	6.95789029	-2.03577891
H	-0.78153619	6.94649784	-0.27924470
C	6.38713812	-0.40901615	-0.77631825
H	6.41457702	-0.41461697	-1.88052032
C	6.66737245	1.01650008	-0.29103713
H	6.52875188	1.04642907	0.80588737
H	5.90195916	1.68881696	-0.71104926
C	8.04672767	1.56921495	-0.63362272
H	8.83002107	0.96516401	-0.14523985
H	8.22228286	1.48174064	-1.72067994
C	8.21126047	3.02729309	-0.21200121
H	7.43417066	3.63451079	-0.70594685
H	8.01902058	3.11087091	0.87099265
C	9.59064442	3.58794361	-0.53631933
H	9.68346586	4.63730873	-0.22552916
H	10.37910121	3.01566093	-0.02529841
H	9.79199138	3.53888028	-1.61682250
C	-4.47498492	2.45791502	-0.26089389
H	-5.07734491	2.75118025	0.61850699
C	-5.24843429	2.85029934	-1.52503496
H	-6.22274323	2.33016411	-1.51768041
H	-4.69944238	2.46604143	-2.40344004
C	-5.49038880	4.34445756	-1.71125825
H	-5.97316916	4.75774734	-0.80786456
H	-4.52532169	4.86955752	-1.81382466
C	-6.35306921	4.65616821	-2.93142907
H	-7.32347710	4.14296119	-2.82718288
H	-5.87597290	4.22567564	-3.82782985
C	-6.57843078	6.14978874	-3.13295510
H	-5.62317477	6.67763600	-3.27242938
H	-7.20315500	6.34891258	-4.01412414
H	-7.07882750	6.59399298	-2.25963285
C	7.40428178	-1.44950974	-0.26841093
H	6.88482086	-2.40062105	-0.05942201
H	7.80671689	-1.11176009	0.70145906

C	8.54161847	-1.73271716	-1.24563460
H	9.25151012	-2.45966951	-0.82835421
H	8.14844898	-2.14917150	-2.18521122
H	9.10040768	-0.82080913	-1.49454776
C	-3.09412207	3.13500084	-0.14926190
H	-2.38011036	2.41509677	0.28569857
H	-2.71528944	3.34510538	-1.16407709
C	-3.09000583	4.41014633	0.68949160
H	-3.40790230	4.20117007	1.72309306
H	-2.08568241	4.85607000	0.73231889
H	-3.77592256	5.16738983	0.28609417
C	2.81764823	3.34257719	2.14074999
H	2.59889006	4.38462056	2.41808444
H	3.16144760	2.85655033	3.06892666
C	3.93628239	3.31564735	1.10078346
H	4.14374516	2.29729906	0.74107577
H	3.68347684	3.92592622	0.22114076
H	4.86848449	3.71719695	1.52235836

4. Spectral copies of the unreported products

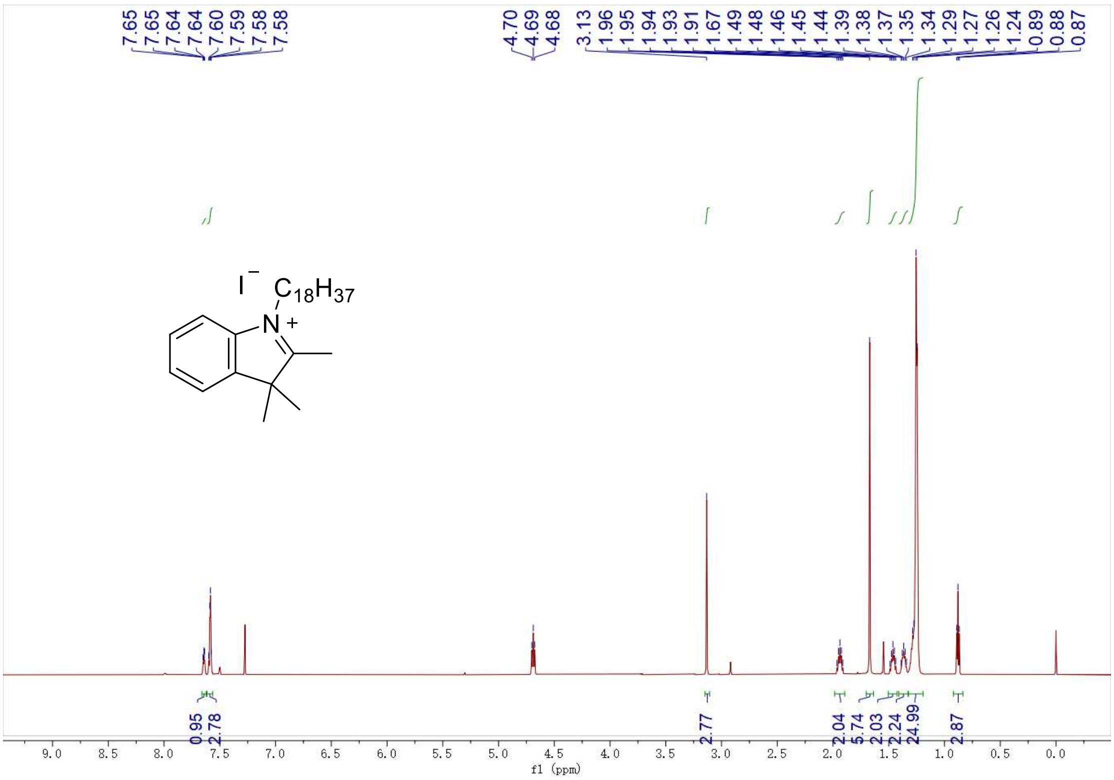

Figure S6. ^{1}H NMR spectra of WB intermediate

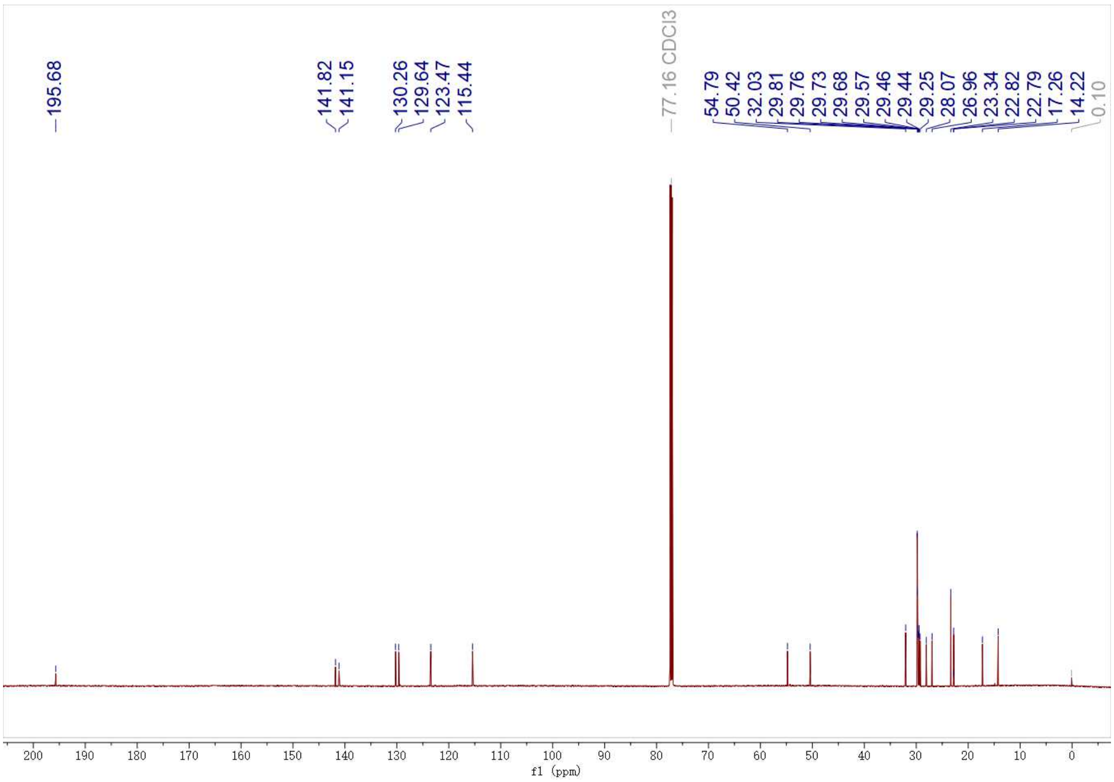

Figure S7. ^{13}C NMR spectra of WB intermediate

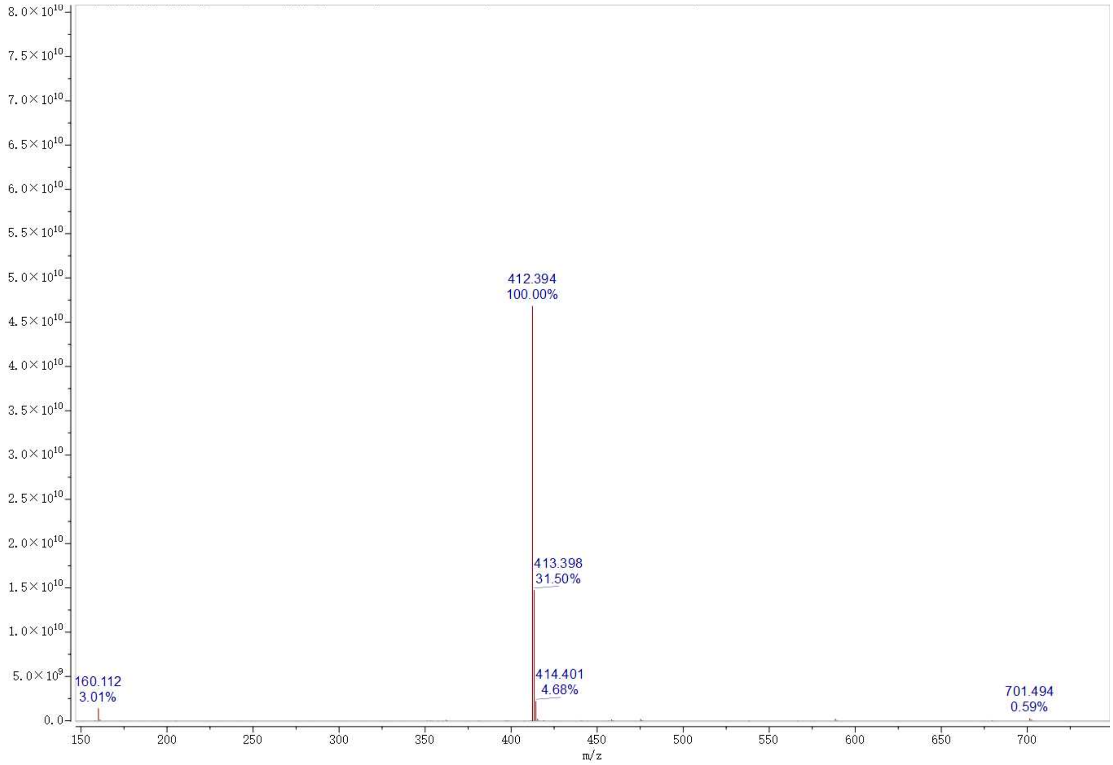

Figure S8. Mass spectra of WB intermediate

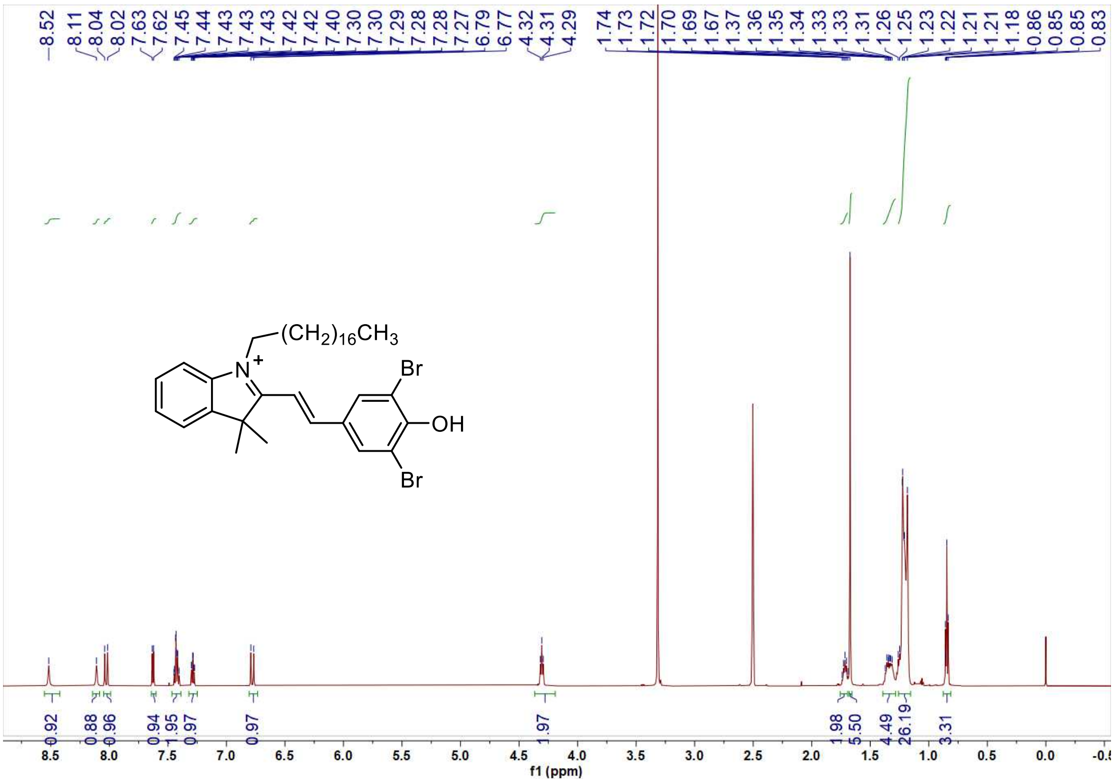

Figure S9. ^{1}H NMR spectra of WB

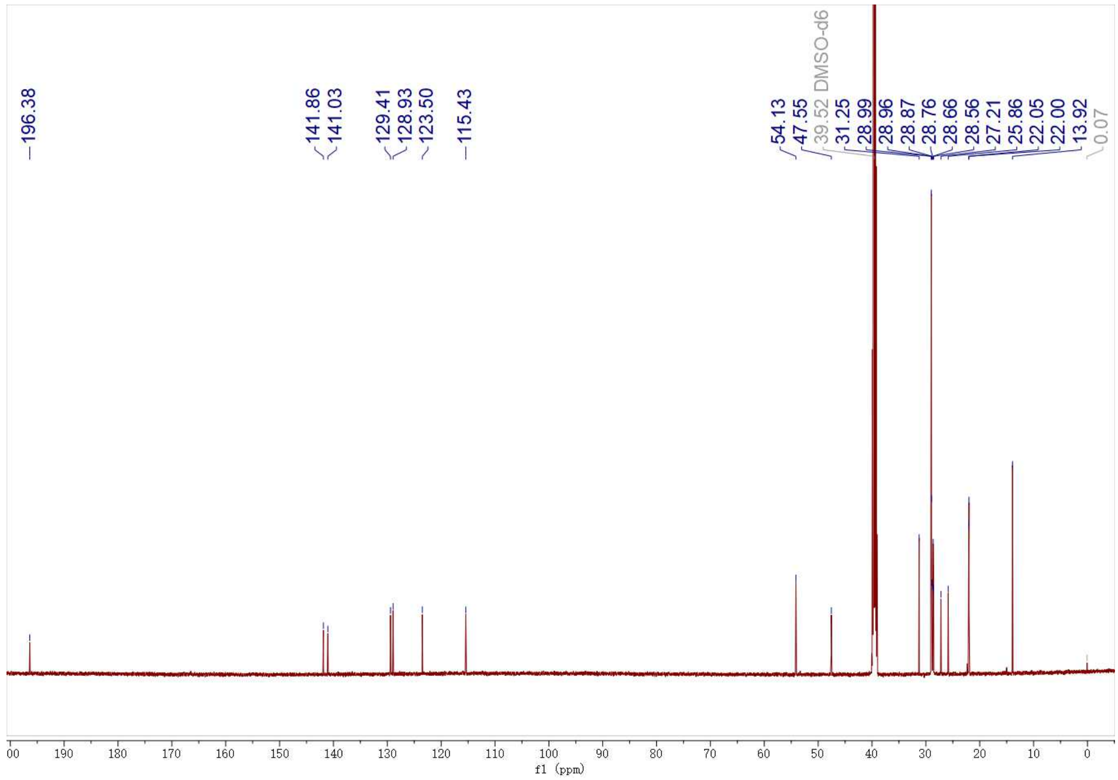

Figure S10. ^{13}C NMR spectra of WB intermediate

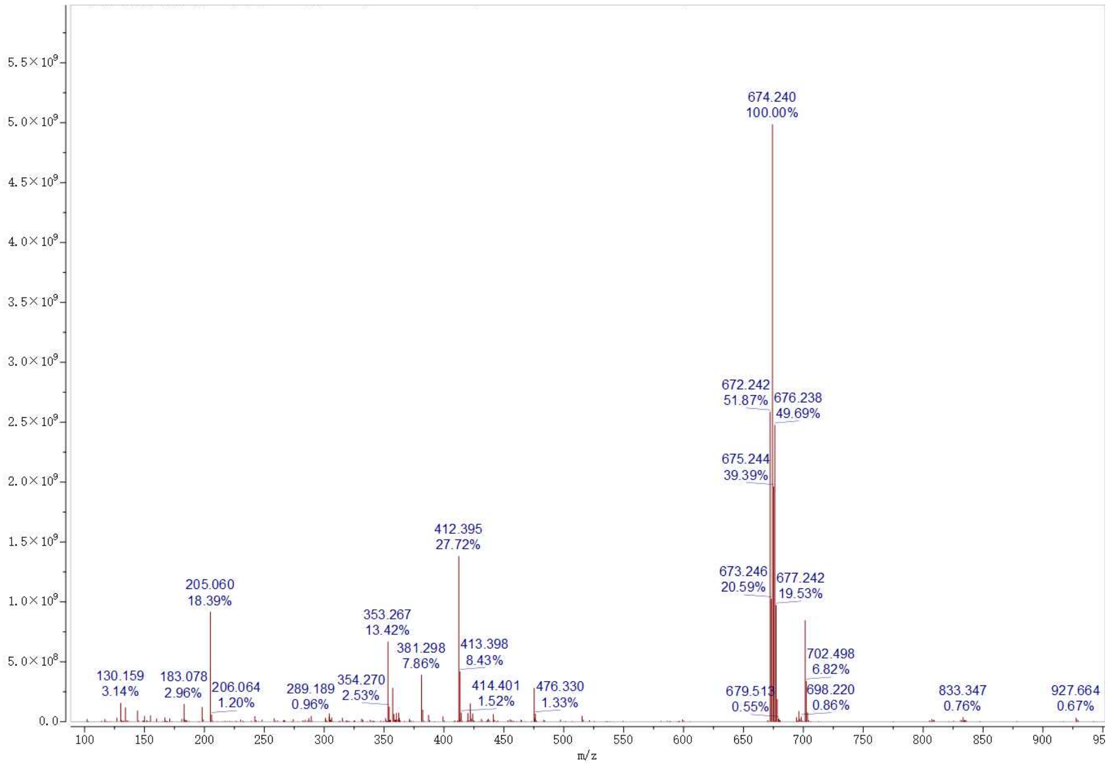

Figure S11. Mass spectra of WB

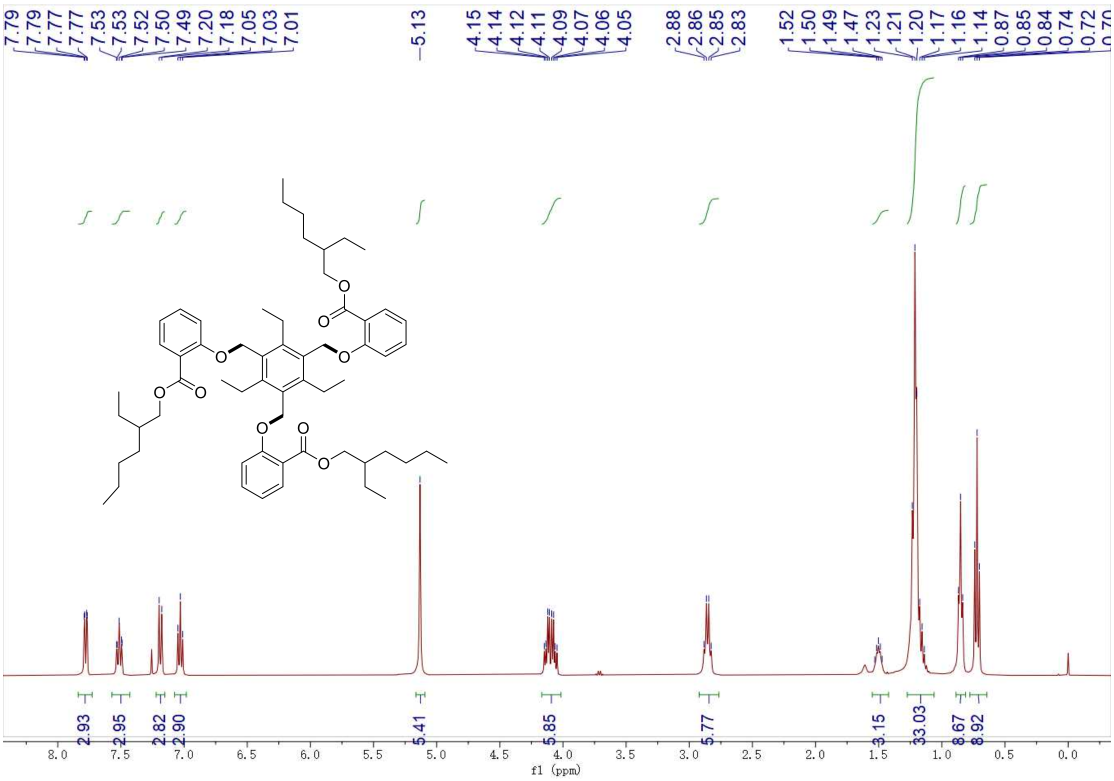

Figure S12. ^{1}H NMR spectra of L

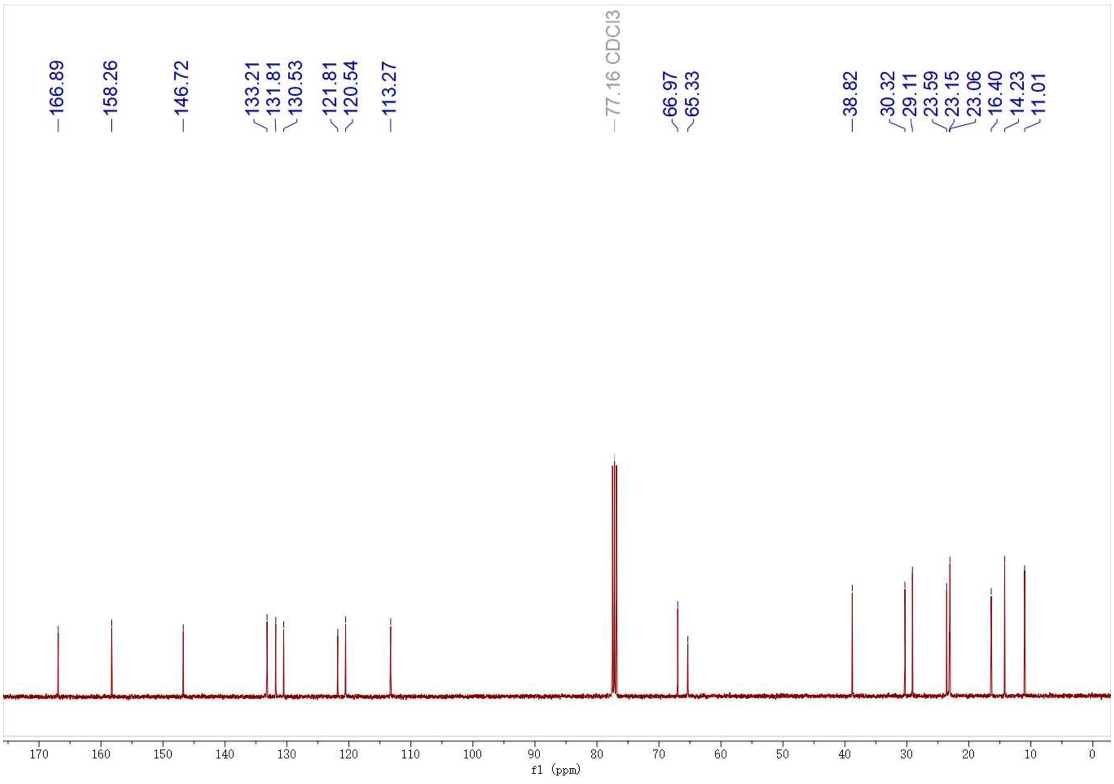

Figure S13. ^{13}C NMR spectra of L

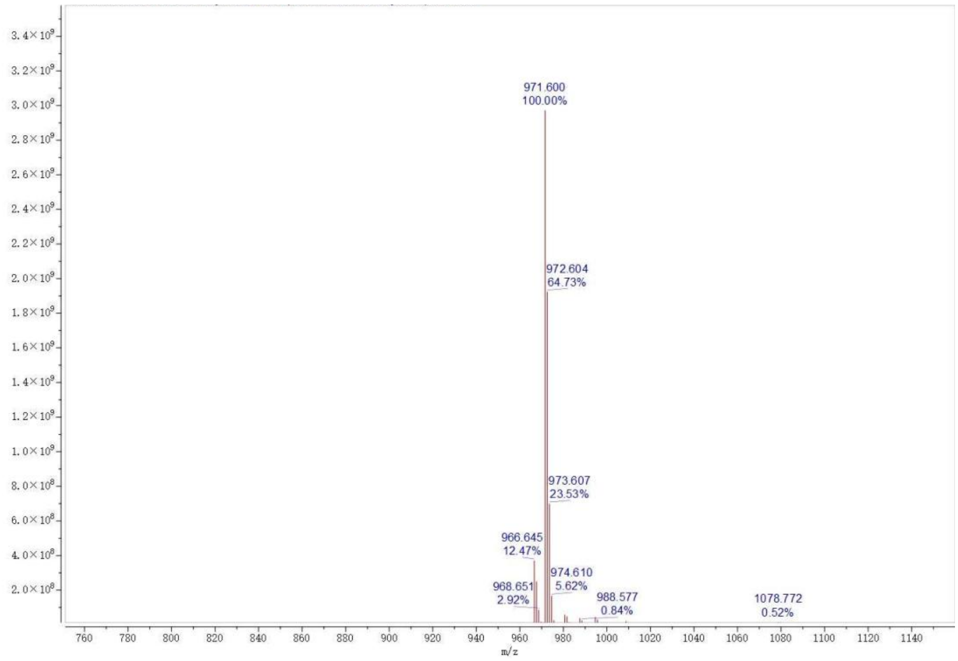

Figure S14. Mass spectra of L

Reference

[1] Gaussian 16, Revision A.03, Frisch, M. J.; Trucks, G. W.; Schlegel, H. B.; Scuseria, G. E.; Robb, M. A.; Cheeseman, J. R.; Scalmani, G.; Barone, V.; Petersson, G. A.; Nakatsuji, H.; Li, X.; Caricato, M.; Marenich, A. V.; Bloino, J.; Janesko, B. G.; Gomperts, R.; Mennucci, B.; Hratchian, H. P.; Ortiz, J. V.; Izmaylov, A. F.; Sonnenberg, J. L.; Williams-Young, D.; Ding, F.; Lipparini, F.; Egidi, F.; Goings, J.; Peng, B.; Petrone, A.; Henderson, T.; Ranasinghe, D.; Zakrzewski, V. G.; Gao, J.; Rega, N.; Zheng, G.; Liang, W.; Hada, M.; Ehara, M.; Toyota, K.; Fukuda, R.; Hasegawa, J.; Ishida, M.; Nakajima, T.; Honda, Y.; Kitao, O.; Nakai, H.; Vreven, T.; Throssell, K.; Montgomery, J. A., Jr.; Peralta, J. E.; Ogliaro, F.; Bearpark, M. J.; Heyd, J. J.; Brothers, E. N.; Kudin, K. N.; Staroverov, V. N.; Keith, T. A.; Kobayashi, R.; Normand, J.; Raghavachari, K.; Rendell, A. P.; Burant, J. C.; Iyengar, S. S.; Tomasi, J.; Cossi, M.; Millam, J. M.; Klene, M.; Adamo, C.; Cammi, R.; Ochterski, J. W.; Martin, R. L.; Morokuma, K.; Farkas, O.; Foresman, J. B.; Fox, D. J. Gaussian, Inc., Wallingford CT, **2016**.

[2] Zhao, Y.; Truhlar, D. G. The M06 suite of density functionals for main group thermochemistry, thermochemical kinetics, noncovalent interactions, excited states, and transition elements: two new functionals and systematic testing of four M06-class functionals and 12 other functionals. *Theor. Chem. Acc.* **2008**, 120, 215–241.

[3] Zhao, Y.; Truhlar, D. G. Density functionals with broad applicability in chemistry. *Acc. Chem. Res.* **2008**, 41, 157–167.

[4] Ansgar Schäfer; Christian Huber; Reinhart Ahlrichs. Fully optimized contracted Gaussian basis sets of triple zeta valence quality for atoms Li to Kr. *J. Chem. Phys.* **1994**, 100, 5829–5835.

[5] Knizia, G. Intrinsic atomic orbitals: An unbiased bridge between quantum theory and chemical concepts. *J. Chem. Theory Comput.* **2013**, 9, 4834–4843.
